# Survival in renal cell carcinoma: a randomized evaluation of tamoxifen vs interleukin-2, α-interferon (leucocytic) and tamoxifen – reply

**DOI:** 10.1054/bjoc.1999.0952

**Published:** 1999-12-08


*British Journal of Cancer* (2000) **82**(1), 246–249
© 2000 Cancer Research Campaign
Article no. bjoc.1999.0907

Letters to the Editor

### Survival in renal cell carcinoma: a randomized evaluation of tamoxifen vs interleukin-2, α-interferon (leucocyte) and tamoxifen

**Sir**

The paper by Henriksson and colleagues has methodologic variations leading to important drawbacks and therefore is of limited value in answering the question of whether immunotherapy improves survival.

The authors state that they compare 'one of the present (and presumed best) treatments, interleukin-2, α-interferon and tamoxifen, with a control arm of tamoxifen only' and that they use 'a schedule reported previously by Atzpodien and colleagues' (Atzpodien et al, 1990). There is a dramatic and probably important difference in the doses of interleukin-2 (IL-2) and α-interferon (IFN-α) between the two protocols. For example, Atzpodien and colleagues gave a total of 36 million IU IL-2/day for 2 days to start their protocol, whereas the Swedish study protocol called for 9 million IU IL-2/day. In general, doses used in the Swedish protocol are 50% of the cited schedule or less. In addition, the authors state that only 40 patients of the 65 patients in the treatment arm received 75% or more of the intended dose; 25 of the patients received less than 75% of the intended dose and five of them less than 25% of the intended dose. No data are available how many patients received the intended dose or at least 90% of it.

It cannot be expected that cytokine effects are completely dose-independent as it seems to be assumed by the authors. The treatment schedule of this protocol constitutes a very different schedule from the one they cite, and substantial dose reduction might decrease therapeutic effects substantially. This treatment variant could be inefficient in improving survival. However, it has to be questioned even whether this is answered by the study. Relevant patient information is missing. No information is available whether only patients with proven progressive disease were included in the study. Generally accepted and well-known risk factors on survival published by Elson such as weight loss, ECOG, or diagnostic time interval (Elson et al, 1988), are not used to stratify the study population of the seven institutions involved in treating the patients. Instead the authors use laboratory data and an evaluation of specific time frames. However, the laboratory data (haemoglobin, platelets, white-cell count, creatinine and albumin) and the time frame analysis have not been shown to substitute for Elson's risk factors in determining survival as assumed by the authors.

Important methodological issues, such as significant dose reduction, are not stated in the Abstract or Methods section and are not discussed. Treatment protocol is given as a legend only and recognition of these important dose modifications in the Abstract is not possible and in the paper needs detailed search. The paper contains little information on the effect of IL-2, IFN-α treatment on survival in progressive metastatic renal cell cancer. A major concern of the authors was toxicity of treatment. In fact, they state that 'immunological manipulation is associated with toxicity, at least during the treatment period'. This is true only for systemic applications. Local applications of cytokines can have immunomodulatory effects and no toxicity at all, as we have shown previously (Huland and Huland, 1989). Inhaled, local IL-2 has also been used with only moderate toxicity by our group (Huland et al 1992, 1997) resulting in stable quality of life during mean 13, 4 months of treatment (Heinzer et al, 1999). Survival data in patients treated with inhalation of IL-2 are promising, compared to risk factors on survival, published by Elson. Local treatment schedules without toxicity might allow efficient therapies to be found without the limitations of dose-dependent systemic toxicity.

*E Huland and H Heinzer*
*Department of Urology, University Hospital Eppendorf, Martinistr. 52, 22046 Hamburg, Germany*

### REFERENCES

Atzpodien J, Körfer A, Franks CR, Poliwoda H and Kirchner H (1990) Home therapy with recombinant interleukin-2 and interferon-2β in advanced human malignancies. *Lancet* **335**: 1509–1512

Elson PJ, Witt RS and Trump DL (1988) Diagnostic factors for survival in patients with recurrence of metastatic renal cell carcinoma. *J Cancer Res* **48**: 7310–7313

Heinzer H, Mir TS, Huland E, Huland H (1999) Subjective and objective prospective long-term analysis of quality of life during inhaled interleukin-2 immunotherapy. *J Clin Oncol* (in press)

Huland E and Huland H (1989) Local continuous high-dose interleukin-2: a new therapeutic model for the treatment of advanced bladder carcinoma. *Cancer Res* **49**: 5469–5474

Huland E, Huland H and Heinzer H (1992) Interleukin-2 by inhalation. Local therapy for metastatic renal cell carcinoma. *J Urol* **147**: 344–348

Huland E, Heinzer H, Mir T and Huland H (1997) Inhaled interleukin-2 therapy in pulmonary metastatic renal cell carcinoma: six years of experience. *Cancer J Sci Am* **3:** 98–105

### Survival in renal cell carcinoma: a randomized evaluation of tamoxifen vs interleukin-2, α-interferon (leucocytic) and tamoxifen – reply

**Sir**

Although the comments by Drs Huland and Heinzer regarding our study comparing tamoxifen versus interleukin-2, α-interferon (leucocyte) and tamoxifen in general terms are acknowledged, it is obvious that there is a need for clarification.

We are well aware that our study could be questioned for various reasons, as directly and straightforwardly outlined in the discussion of the article. Nevertheless, we are somewhat astonished by the reason for the critical comments by Drs Huland and Heinzer, related for the most part to the fact that they only rely their discussion on non-randomized studies including selected population of patients. In fact, we lack references to well controlled studies. An obvious shortcoming of most of the previously published studies within the field of immunotherapy is the conclusion that the response rate was superior to 'historic controls', although these trials were not randomized, and did not use case-matched controls (Philip et al, 1993).

It has to be emphasized that our study investigated the general applicability of the therapy concept in the management of patients with good performance status (WHO 0–2). Even if it is a possibility that the doses delivered to the patients are lower than proposed to be optimal by Huland and Heinzer, it was clear that in our hands many of the patients (note patients with high performance status) required dose-reduction to be able to manage the treatment at all. The toxicity encountered was in accordance with earlier reported, and included a substantial number of patients with grade 3 and 4 toxicity. Thus, the adverse effects seen deteriorated quality of life in a significant manner of the patients treated with immunotherapy. Publication is also underway for the final evaluation of quality of life for the whole study. This gives at least a further support that the doses used were of clinical significance, i.e. the toxicity was substantial. There exist no conclusive studies that clearly demonstrate a dose–response relationship for cytokines in the clinical situation. Most likely, there is a quite different dose–response correlation for cytokines ('bell-shaped') compared to chemotherapeutics. Thus, it is not clear that 'more is better'.

The survival in our study was similar in the two groups of patients, even in respect to long-term survival. Moreover, the survival was quite comparable with survival data reported in other studies of renal cell carcinoma patients treated with IL-2/α-IFN (e.g. Facendola et al, 1995). In fact, median survival in each of the treatment arms was better than that seen in Swedish registry studies. This gives further support that the doses delivered to patients were of clinical significance. The survival analysis did not seem to be different regardless of the time frame of the analysis (from the date of primary diagnosis or the start of the treatment or from the time of first signs of metastatic spread).

There was no obvious initial variation in laboratory parameters or metastatic spread of the disease, which further reduces the risk of differences in prognostic factors between the groups. Furthermore, there exist several previously published studies that support our observations (Steineck et al, 1990; Wagstaff et al, 1995; Ljungberg and Henriksson, 1997).

The past decades have without doubt shown an outstanding increase in the knowledge about tumour immunology and biotherapy. In renal cell carcinoma, the relatively high response figures induced by a biotherapy approach have encouraged extensive clinical studies. We would like to stress that we do not deny the beneficial effects of biotherapy seen by several other authors (e.g. Atzpodien et al, 1995), and agree that there might exist subgroup of highly selected patients with renal cell carcinoma that can really benefit from biotherapy. We also agree that other treatment approaches, like inhalation of IL-2, can be promising. Therefore, we are eagerly waiting the first reported experiences from 1989 of local delivery of IL-2 by Huland and co-workers evaluated in a controlled randomized study.

At present, regardless of our study, there is no standard immunotherapy (a conclusion made in our study) that can be recommended since the results obtained do not suggest an obvious therapeutic benefit for the larger patient population suffering from advanced renal cell carcinoma. It is obvious that there is much need for investigation to find the optimal biotherapy schedule, i.e. a significant increase in survival with an acceptable quality of life.

*R Henriksson*
*Department of Oncology, University Hospital, Umeå, Sweden,*
*P Wersäll*
*Radiumhemmet, Karolinska Institute, Stockholm, Sweden*

**REFERENCES**

Atzpodien J, Hänninen LE, Kirchner H, Bodenstein H, Pfreundschuh M, Rebmann U, Metzner B, Illiger H-J, Jakse G, Niesel T, Scholz H-J, Wilhelm S, Pielmeier T, Zabrewski G, Blum G, Beier J, Müller G-W, Duensing S, Anton P, Allhoff E, Jonas U and Poliwoda H (1995) Multiinstitutional home-therapy trial of recombinant human interleukin-2 and interferon alfa-2 in progressive metastatic renal cell carcinoma. *J Clin Oncol* **13**: 497–501

Facendola G, Locatelli MC, Pizzocaro G, Piva L, Pegoraro C, Bobbio Pallavicini E, Signaroldi A, Meregalli M, Lombaardi F, Beretta GD, Scanzi F, Labianca R and Luporini G (1995) Subcutaneous administration of interleukin 2 and interferon-alpha-2b in advanced renal cell carcinoma: a confirmatory study. *Br J Cancer* **72**: 1531–1535

Huland E and Huland H (1989) Locla continuous high-dose interleukin-2: a new therapeutic model for the treatment of advanced bladder carcinoma. *Cancer Res* **49**: 5469–5474

Ljungberg B and Henriksson R (1997) Immunotherapy of metastatic renal cell carcinoma. *Curr Opin Urol* **7:** 252–258

Philip T, Negrier S, Lasset C, Coronel B, Bret M, Baly JY, Merrouche Y, Carrie C, Kaemmerlein P, Chauvin F, Favrot M, Oskam R, Tabah I, Clavel M, Moskovtchenko JF and Mercatello A (1993) Patients with metastatic renal carcinoma candidate for immunotherapy with cytokines. Analysis of a single institutional study on 181 patients. *Br J Cancer* **68**: 1036–1042

Steinec G, Strander H, Carbin B-E, Borgström E, Wallin L, Achtnich U, Arvidsson A, Söderlund V, Näslund I, Esposti P-L and Norell SE (1990) Recombinant leukocyte interferon alpha-2a and medroxyprogesterone in advanced renal cell carcinoma. A randomized trial. *Acta Oncol* **29**: 155–162

Wagstaff J, Baars JW, Wobink G-J, Hoekman K, Eerenberg-Belmer AJM and Hack CE (1995) Renal cell carcinoma and interleukin-2. A review. *Eur J Cancer* **31A**: 401–408

### Hereditary factors in basal cell carcinoma of the skin: a population-based cohort study in twins

**Sir**,

Milan et al (*Br J Cancer* 1998 **78**: 1516–1520) reported on a large twin study investigating the contribution of hereditary factors in basal cell carcinoma (BCC) in Finland. This twin study based on 12 941 adult twin pairs with 43 years follow-up data concluded that genetic factors are not necessary to explain the distribution of BCC in twins. These findings are of major importance; however, a number of points need to be addressed in the analysis before these conclusions can be accepted.

© 2000 Cancer Research Campaign

